# Chemokines from a Structural Perspective

**DOI:** 10.3390/ijms18102088

**Published:** 2017-10-02

**Authors:** Michelle C. Miller, Kevin H. Mayo

**Affiliations:** Department of Biochemistry, Molecular Biology & Biophysics, University of Minnesota, Minneapolis, MN 55455, USA; mill0935@umn.edu

**Keywords:** chemokine, structure, NMR, heterodimers, interactome

## Abstract

Chemokines are a family of small, highly conserved cytokines that mediate various biological processes, including chemotaxis, hematopoiesis, and angiogenesis, and that function by interacting with cell surface G-Protein Coupled Receptors (GPCRs). Because of their significant involvement in various biological functions and pathologies, chemokines and their receptors have been the focus of therapeutic discovery for clinical intervention. There are several sub-families of chemokines (e.g., CXC, CC, C, and CX3C) defined by the positions of sequentially conserved cysteine residues. Even though all chemokines also have a highly conserved, three-stranded β-sheet/α-helix tertiary structural fold, their quarternary structures vary significantly with their sub-family. Moreover, their conserved tertiary structures allow for subunit swapping within and between sub-family members, thus promoting the concept of a “chemokine interactome”. This review is focused on structural aspects of CXC and CC chemokines, their functional synergy and ability to form heterodimers within the chemokine interactome, and some recent developments in structure-based chemokine-targeted drug discovery.

## 1. Chemokine Structures

**Figure 1:**
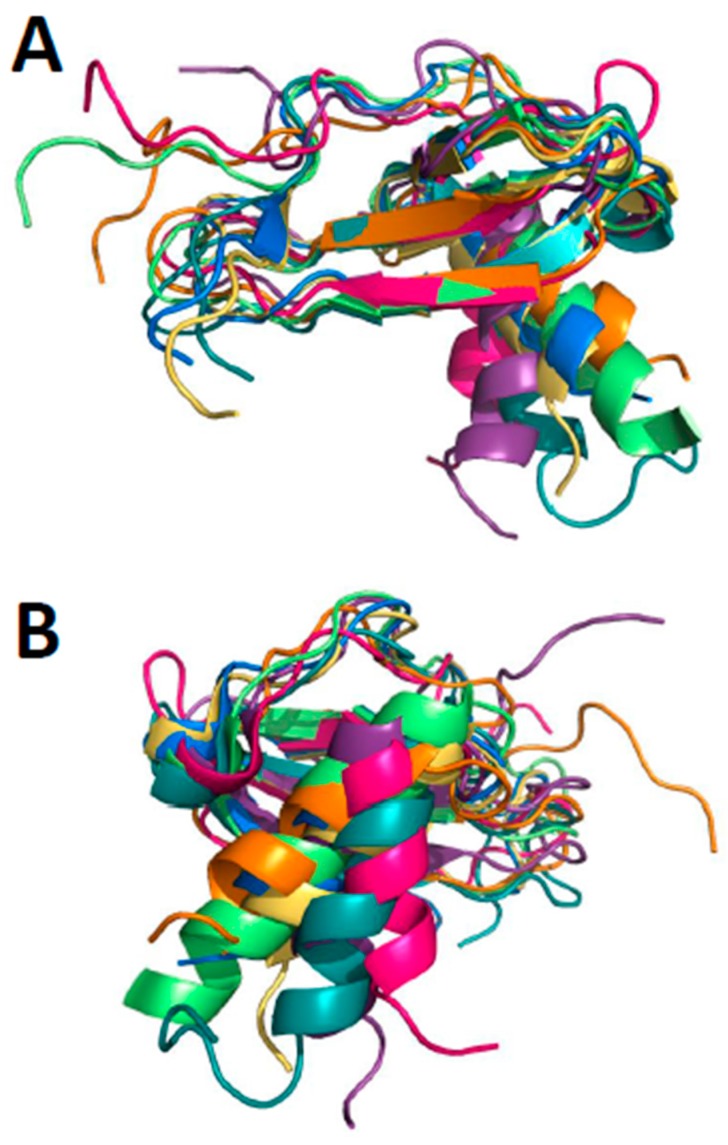
Superposition of seven monomer subunits from reported structures of CXC and CC chemokine homodimers is shown: CXCL4 M2 variant (Protein Data Bank, PDB: 1PFM), CXCL8 (PDB: 1IL8), CXCL12 (PDB: 3HP3), CXCL13 (PDB: 4ZAI), CCL5 (PDB: 5COY), CCL14 (PDB: 2Q8R), and CCL20 (PDB: 1HA6). (**A**) Only atoms within the three-stranded β-sheet are superimposed with RMSD values ranging between ~1.3 and ~1.7 Å; (**B**) Superimposed structures shown in panel A are rotated by about 180° to illustrate how C-terminal helices are folded onto the β-sheet at somewhat different angles.

Chemokines are a family of small, highly conserved proteins (8 to 12 kDa) involved in many biological processes, including chemotaxis [1], leukocyte degranulation [2], hematopoiesis [3], and angiogenesis [4,5]. Chemokines are usually categorized into sub-families based on the sequential positioning of the first two of four highly conserved cysteine residues: CXC, CC, and CX3C [6]. The C chemokine sub-family is the exception, with only one N-terminal cysteine residue. In the largest subfamilies, CC and CXC, the first two cysteines are adjacent (CC motif) or separated by one amino acid residue (CXC motif). C type chemokines lack the first and third of these cysteines, and CX3C chemokines have three amino acids between the first two cysteine residues. Even though sequence identity between chemokines varies from about 20% to 90%, their sequences overall are highly conserved. Nevertheless, all chemokines adopt essentially the same fold as illustrated in Figure 1 with the superposition of seven chemokines (monomer units): CXCL4, CXCL8, CXCL12, CXCL13, CCL5, CCL14, and CCL20. These structures all consist of a flexible N-terminus and N-terminal loop, followed by a three-stranded antiparallel β-sheet on to which is folded a C-terminal α-helix [7], exemplified early on by CXCL4 [8], CXCL7 [9], CXCL8 [10], and CCL2 [11]. Only atoms within the three-stranded β-sheet have been superimposed (Figure 1A), and RMSD values for backbone atoms of these β-strands range between ~1.3 and ~1.7 Å, with loops being more variable due in part to increased flexibility and differences in amino acid type and number of residues. Note that when the β strands are superimposed, the C-terminal helices are folded onto the β-sheet at somewhat different angles (Figure 1B). The highly conserved cysteine residues (four in CXC and CC chemokines) pair up to form disulfide bridges that are crucial to maintaining structural integrity, which is a prerequisite for chemokine binding to their respective GPCRs [12].

**Figure 2:**
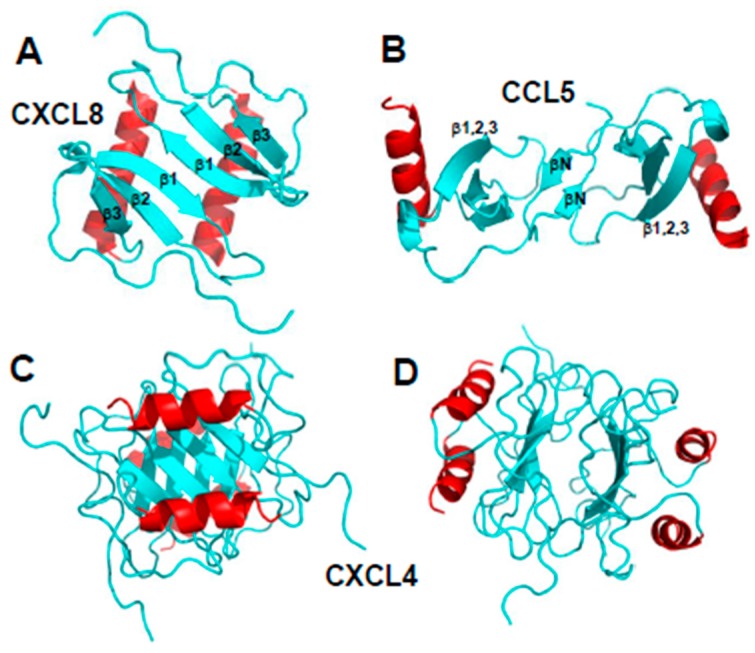
Structures of CXC chemokine CXCL8 (Interleukin-8, PDB access code 1IL8, [10]) (panel **A**) and CC chemokine CCL5 (RANTES, PDB access code 5COY, [17]) (panel **B**) are shown. Two orientations of the CXCL4 M2 tetramer structure (platelet factor-4, PF4; PDB access code 1PFM, [18]) are shown in panels (**C,D**). C-terminal helices are colored red, and the remaining sequences are colored cyan.

Chemokine monomers usually associate to form oligomers, primarily dimers, but some are also known to form tetramers [13,14] and higher-order species, e.g., [15,16]. Despite their highly conserved monomer structures, chemokines form different types of oligomer structures depending on the sub-family to which they belong [7]. Within each chemokine sub-family, dimer structures are essentially the same. Figure 2A,B illustrates the dimer structures for CXC chemokine CXCL8 (Interleukin-8 [10]) and CC chemokine CCL5 (RANTES [17]). The more globular CXC-type dimer is formed by interactions between β1 strands from each monomer subunit that extends the three stranded anti-parallel β-sheet from each monomer into a six-stranded β-sheet, on top of which are folded the two C-terminal α-helices, running antiparallel (Figure 2A). On the other hand, CC-type chemokines form elongated end-to end type dimers through contacts between short N-terminal β-strands (labeled βN) with the two C-terminal helices running almost perpendicular to each other on opposite sides of the molecule (Figure 2B). Nevertheless, some CC-type dimer structures like CCL5 have been reported to differ in the relative orientation of some secondary structure elements (e.g., C-terminal α-helices), which may be related to differences in structural dynamics and/or crystal lattice effects [15].

An example of a chemokine tetramer formation is shown in Figure 2C,D with the structure of CXCL4 M2 variant (platelet factor-4, [18]). In this example, two CXC-type dimers associate to form a β-sandwich, with the β-sheet of one dimer lying on top of the β-sheet of the other dimer (Figure 2C). The β-sandwich is rotated by ~90° in Figure 2D to better illustrate the contacts between β-sheets and show the center of the CXCL4 tetramer structure. Tetramers have also been observed for other chemokines, e.g., CXCL7 [19]. Moreover, the tetramer structures of different chemokines can vary considerably. For example, two distinct tetramers were reported for CXCL10 [20], one having both CC- and CXC-type dimer topologies and displaying an entirely new conformation. Furthermore, comparison of CXCL4 tetramers [21] and CCL2 tetramers [22] show that they both display CC- and CXC-type dimer motifs.

**Figure 3:**
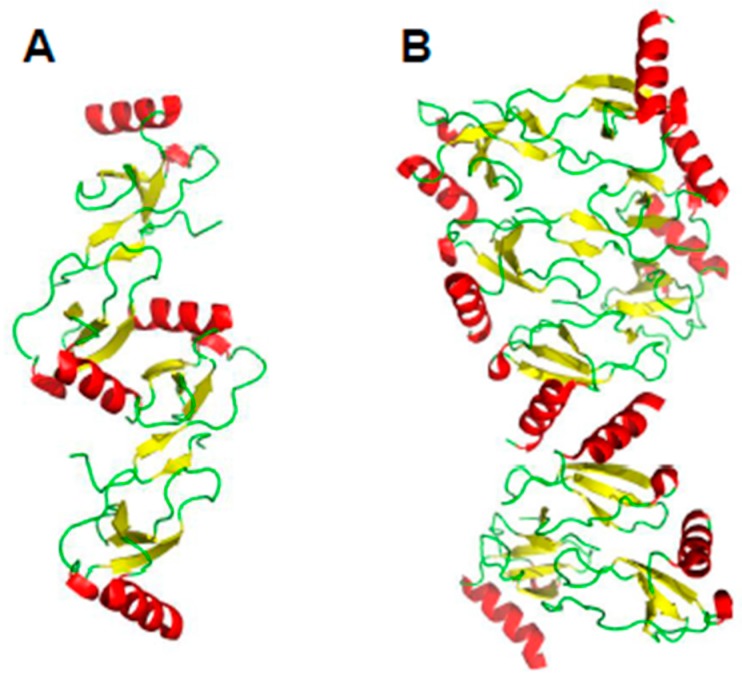
Higher-order oligomer structures of CCL5. (**A**) Proposed oligomer structure of CCL5 deduced from analysis of NMR, MS, and SAXS data (PDB access code: 2L9H, [15]; (**B**) Modeled oligomer structure of CCL3 (PDB access code: 5L2U, [17]) is shown to illustrate how a CC-type dimer could associate in a somewhat different fashion into a higher order oligomer. C-terminal helices are colored red; β-strands are colored yellow, and aperiodic sequences/loops are colored green.

In other instances, higher order oligomer chemokine structures have not been observed, e.g., CXCL8 or CXCL1 (Growth-related protein-α, Gro-α) (e.g., [23]), but this may be due to the presence of only very low oligomer populations and limitations of experimental techniques used to investigate them. Some CC-type chemokines are not known to form tetramers, yet they do associate to form higher-order oligomers, like CCL5 [15] and CCL27 [16]. Figure 3A illustrates a proposed oligomer structure of CCL5 deduced from analysis of NMR, MS, and SAXS data [15]. This model shows the CCL5 oligomers consisting of chemokine CC-type dimers assemble in a linear fashion. Figure 3B shows the modeled oligomer structure of CCL3 [17], which shows how a CC-type dimer can associate in a somewhat different fashion into a higher order oligomer. Aside from standard CXC- and CC-type inter-subunit interactions seen in the CCL3 oligomer structure (Figure 3B, [17], energetically favorable interactions are also observed between the post-β3 loop of one subunit (Thr43-Arg44-Lys45 and Arg47) and the helix (Glu66) and β1 strand (Glu26-Try27) of the opposing subunit, respectively, suggesting that other types of heterodimers may associate in situ. Nevertheless, whereas these studies underscore the importance of the chemokine CC-type dimer as a building block for larger chemokine oligomers, the idea of a single, well-described dimer structure defining the topology of larger oligomers may be too simplistic.

Chemokine monomers, dimers, and higher-order oligomers exist in a complex equilibrium where distinct oligomer structures co-exist and interconvert within a dynamic distribution [15,21,24,25,26,27,28]. For example, distinct co-existing structures have been reported for the monomers and dimers of the chemokines XCL1 and CCL27 [16]. Early on, this was well exemplified by NMR studies on CXCL4 [13,29], low affinity CXCL4 [30], and CXCL7 (platelet basic protein, [19]) and its N-terminal degradation products CTAP-III (connective tissue-activating peptide III) and NAP-2 (neutrophil-activating peptide-2) [31]. In general, the weighting of oligomer populations is dictated by the amino acid composition and conformation of inter-subunit interfaces [32]. This in turn determines the thermodynamic stability of the complexes, with some chemokines forming stronger oligomers and others much weaker ones or remaining as monomers/dimers. This equilibrium distribution can be perturbed by changing solution conditions (e.g., lower pH, buffer type, ionic strength, presence of ligands like heparin) as reported e.g., with CXCL4 [13,29,33], CXCL7 [34], CXCL12 [35,36], and CCL11 [37]. Relatedly, oligomer subunit exchange is the primary reason why not all chemokines can be crystallized or why their structures cannot be solved using NMR spectroscopy.

## 2. Chemokine Heterodimers

Because chemokine monomer structures are highly conserved, chemokine quarternary structures are determined primarily by the amino acid residues present at the particular inter-subunit interface [32]. Therefore, monomers of different chemokines may be swapped if the arrangement and composition of residues at a given monomer–monomer interface in a heterodimer make for an energetically favorable state relative to that in either homodimer. Indeed, Guan et al. [38] demonstrated that CC chemokines CCL3/4 and CCL2/8 (macrophage inflammatory protein 1α (MIP-1α) and 1β (MIP-1β), respectively) form heterodimers in vitro, as well as being secreted as heterodimers from activated human monocytes and peripheral blood lymphocytes, suggesting that this CC chemokine-based heterodimer may impact on intracellular signaling via binding to, and activation of, its receptor CCR5 [38]. It has also been shown that at least three members of the CXC chemokine sub-family, CXCL4, its *N*-terminal chimera PF4-M2 [18], and CXCL8, readily exchange subunits to form heterodimers that exhibit similar equilibrium dimerization constants (*K*_d_) as observed for homodimers [39,40]. CXC and CC chemokine heterodimers (i.e., CXCL4 and CCL5) were also shown to form in cells in culture, as well as in vivo [41,42]. 

**Figure 4:**
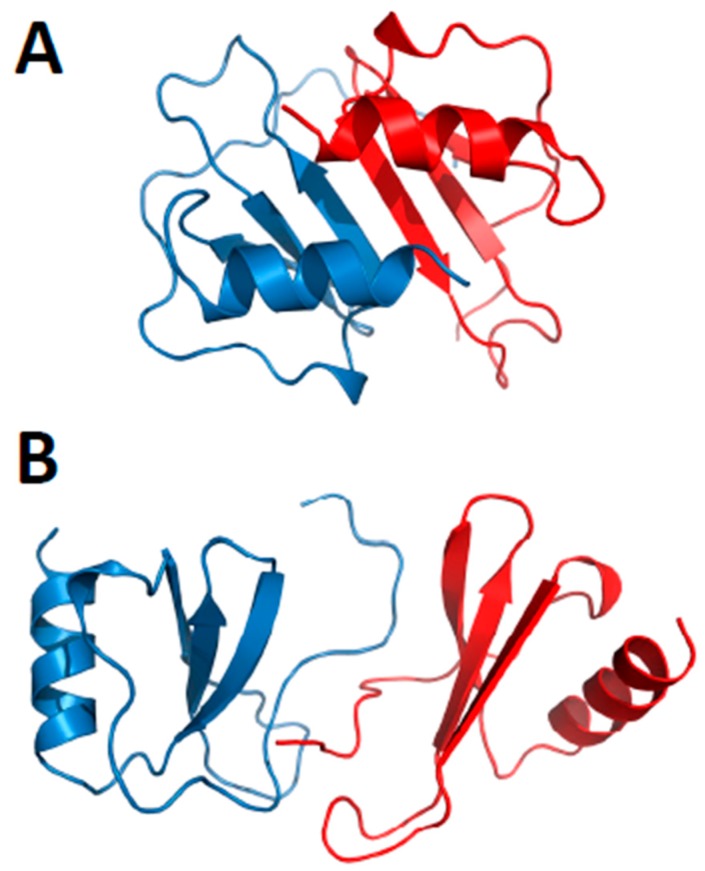
Modeled structures of CXCL4/CCL5 heterodimers. These structures are based on NMR chemical shift and intensity changes from HSQC experiments on CXCL4 and CCL5. NMR data were used to direct manual docking and energy minimization using Molecular Dynamics (MD) simulations, as discussed in von Hundelshausen et al. [42]. MD simulations and energy minimization were done with CCL5 and CXCL4 monomer subunits initially docked as a CXC-type dimer (**A**) or a CC-type dimer (**B**), with the CC-type heterodimer being energetically favored. CXCL4 monomer subunits are shown in red, and CCL5 monomer subunits are shown in blue. These structures were produced by Dr. Kanin Wichapong, Maastricht University, The Netherlands.

Nesmelova et al. [32] explored the energetic basis for heterodimerization of CXC and CC chemokines by using molecular mechanics and the Poisson–Boltzmann surface area (MM-PBSA) approach to calculate binding free energies and to predict which pairs of CXC and CC chemokines would likely form in solution. This study indicated that heterodimers within and between members of CXC and CC sub-families can occur. Calculations were done to assess also which type of heterodimer might form, i.e., CXC-type vs. CC-type heterodimers. In this regard, it was reported that CXCL4 could make thermodynamically favorable interactions with CXCL1, CXCL7, and CXCL8, as well as CXCL1/L8, CXCL7/L8 and CXCL1/L7, with all pairs forming only CXC-type dimers. CC chemokine CCL2 could also favorably pair up with CCL5 and CCL8, with CXC-type heterodimers being favored with the CCL2/CCL8 pair. Several CXC/CC mixed chemokine pairs were also examined, with CCL2/CXCL4 and CCL2/CXCL8 favoring CXC-type heterodimer formation, and CCL5/CXCL4 greatly favoring the CC-type dimer, and CXCL8/CCL5 forming either equally well. Modeled structures of the CCXL4/CCL5 heterodimer are shown in Figure 4A,B for both CXC- and CC-type dimers, with the CC-type heterodimer being highly energetically favored over the CXC-type heterodimer. Some of these, like the CXCL4/CCL5 heterodimer, have been experimentally validated in vitro and/or in vivo [42].

Chemokines bind strongly and specifically to glycosaminoglycans (GAGs) [27,43,44,45,46] that are comprised of sulfated LacNAc disaccharide repeat units and can vary in chain length and sulfation pattern. For example, CCL5 homo-dimers interact with GAGs in a specific manner [47,48,49], with GAG binding affinity depending on both the type of GAG and its sulfation pattern [49]. This has also been reported for CCL2 [50] and CCL11 [43]. In general, highly negatively charged GAGs interact electrostatically with positively charged amino acid residues in chemokines. Contrary to some CXCL4/GAG binding models, which center around the cluster of lysines within the chemokine C-terminal α-helix, Mayo et al. [51] used NMR and site-directed mutagenesis to demonstrate that the loop containing Arg20, Arg22, His23 and Thr25, as well as Lys46 and Arg49, play a greater role in GAG/heparin binding. Moreover, even though electrostatic interactions are understood to play a key role in GAG binding to chemokines, other forces also contribute to their binding specificity (e.g., CXCL4, [51] and XCL1, [52]).

Chemokine homo-oligomers have also been known for some time for their ability to bind relatively strongly to GAGs [48], and conversely, binding to GAGs can induce chemokine homo-oligomer formation, as exemplified by CCL5 homo-oligomer formation [27,53,54]. GAG binding can also have a significant effect on chemokine structure [27,50], structural dynamics [47], homo-oligomerization [55], and chemokine receptor dimerization, e.g., CCR2 [56]. For example, Rek et al. [27] reported that CCL5 undergoes a structural transition upon GAG binding, and Verkaar et al. [57] found that GAG binding affects chemokine cooperativity. Furthermore, Mikhailov et al. [23] demonstrated early on that heparin dodecasaccharide binding to CXCL4 induces higher-order oligomer formation that is dependent upon the chemokine:GAG molar ratio.

Chemokine hetero-oligomers are also stabilized by binding to GAGs [24]. Crown et al. [56] characterized the effects of GAG binding on heterodimerization of CCR2 ligands CCL2 (MCP-1), CCL8 (MCP-2), CCL7 (MCP-3), CCL13 (MCP-4), and CCL11 (eotaxin). These authors reported that CCL2 and CCL8 form strong and specific CC-type heterodimers, whereas CCL2/CCL13, CCL2/CCL11, and CCL8/CCL13 heterodimers are only moderately stable, and CCL7 did not form heterodimers with any other CCR2 chemokine ligand. Moreover, heterodimer formation was enhanced by chemokine binding to GAGs (heparin pentasaccharide, Arixtra). In their study, Arixtra promoted formation of CCL8/CCL11 and CCL2/CCL11 heterodimers, which otherwise either did not form or formed only weakly.

## 3. Functional Impact of Chemokine Structure

Chemokines play a significant role in biology and are involved in many pathologic disorders, including cancer, HIV/AIDS, and atherosclerosis [58,59,60]. About fifty chemokines are involved in various aspects of cell interactions and communication with the immune system. In general, chemokines trigger their functional activities by binding to cell surface G-protein coupled receptors (GPCRs) [61,62,63,64]. For example, CXCL12 binds to and activates the CXCR4 receptor; CXCL7 (platlet basic protein and its *N*-terminal degradation product NAP-2) signals through both CXCR1 and CXCR2 receptors, and CXCL9, CXCL10 and CXCL11 work through CXCR3 receptor [65].

The best way to understand how chemokines function on their respective receptors is through a structural knowledge of the chemokine ligand/receptor complex [66]. However, the determination of high-resolution structures of chemokine ligand/receptor complexes is highly challenging, and no such structures are presently available. Nevertheless, structural biology plays a major role in delineating how chemokines interact with their GPCR receptors, which in turn relates to how chemokines trigger cell signaling, information that is crucial to designing chemokine antagonists. Given the size of GPCRs and the general difficulties of working with them in vitro, little is known about which residues within any GPCR direct chemokine ligand binding. However, existing evidence indicates that the N termini of some GPCRs are involved in binding chemokine ligands [67,68,69,70,71].

Most studies have focused on defining those residues within the chemokine ligands themselves that are primarily responsible for binding to and activating GPCRs. In particular, the tripeptide ELR sequence (Glu-Leu-Arg) within the dynamic N-terminus of some CXC chemokines was determined to be crucial for interactions with GPCRs. In terms of function, CXC chemokines with the ELR motif (ELR1: CXCL1, 2, 3, 5, 6, 7, 8) generally promote angiogenesis and those lacking the ELR motif (ELR2: CXCL4, 9, 10) have angiostatic properties [5,72]. Residues within the N-loop between the two N-terminal cysteines, as well as in the helix, can also be involved in GPCR binding [14,73]. Even though the Arg-Phe-Phe-Arg-Glu-Ser-His sequence within this region of CXCL12 is important for receptor binding affinity [74], this is not always the case, and a number of residues throughout the surface of other chemokines have been found to be crucial for receptor binding. In CXCL10 and CCL2, the loops between β-strands are also important for high-affinity receptor binding [71,75]. Binding interactions can also vary depending on the chemokine and which GPCR is involved in their interplay.

Recently, Handel et al. and Volkman et al. have taken the lead in studies aimed at defining structurally crucial interactions between chemokine ligands and their respective GPCRs. Hemmerich et al. [71] showed early on, using primarily mutagenesis studies, that the complex between CCR2 and CCL2 was mediated in part by a few basic amino acid residues in CCL2 and acidic residues (particularly a DYDY tyrosine sulfation motif) in CCR2. Their model suggests that the DYDY motif might bind to a basic residue pocket on CCL2. Such electrostatic interactions from acidic and basic side chains that were also found to be important within the CXCR4:vCCL2 complex [76,77]. vCCL2 (viral macrophage inflammatory protein-II) is expressed by human herpes virus-8 and can bind to multiple chemokine receptors, including CCR5 and CXCR4, thus vCCL2 is quite interesting due to its ability to inhibit HIV infection.

Other studies have reported on chemokine ligand interactions with CXCR4 and CCR2. Ziarek et al. [78] merged information on the NMR-derived structure of a constitutively monomeric CXCL12 variant bound to the amino terminus of CXCR4 with a crystal structure of the trans-membrane domain of CXCR4. Their work showed that the CXCL12:CXCR4 interface allowed previously unknown interactions to be identified, which raised questions about the classical “two-site model” for chemokine-receptor recognition. Moreover, the study demonstrated that the CXCR4 contacts with monomeric CXCL12 were different from those made by dimeric CXCL12, which only stimulates GPCR-dependent signaling. 

Using a different approach, Kufareva et al. [79] employed disulfide trapping to identify how chemokines bind their receptors in order to guide molecular modeling. Early attempts in disulfide crosslinking between CXCR4 and CXCL12 were guided by the NMR structure of a CXCL12 dimer in complex with a 38-residue peptide isolated from the N-terminus of CXCR4 (CRS1) [80]. This work with CXCL12 and CXCR4 was recently continued with a combination of computational modeling, functional assays, and biophysical approaches to assess the stoichiometry and geometry of the interaction in this chemokine ligand/receptor pair [81]. In fact, their cysteine trapping experiments allowed residue proximities to be derived enabling construction and validation of a 1:1 receptor:chemokine model consistent with the two-site model of receptor activation and accumulating evidence supporting monomers as the minimal functional unit of binding to GPCRs [81].

Nevertheless, the simple two-site model in chemokine receptor signal transduction may be inadequate to explain chemokine function in all instances, and new paradigms are required [82]. For example, chemokine monomers and homodimers can interact with and activate their cell surface receptors somewhat differently, as exemplified with CXCL12 where its oligomer state directs the inhibition of metastasis through distinct CXCR4 interactions and signaling pathways [83]. Moreover, chemokine activities can be quite varied. For example, while CXCL9 and CXCL10 both have potent antitumor activities through attraction of cytotoxic T lymphocytes and inhibition of angiogenesis, CXCL11 is more potent in terms of its antitumor activity [84]. The modulation of chemokine responses in terms of synergy and cooperativity has been nicely reviewed by Proudfoot et al. [85].

Understanding chemokine structure-function relationships has been complicated by reports that chemokine heterodimers and/or hetero-oligomers can also form and are associated with some cellular responses [25,32,38,39,40,41,42,86,87,88]. This has been demonstrated e.g., with the formation of the CXCL8/CXCL4 heterodimer, which enhances both CXCL4-induced endothelial cell proliferation, and CXCL8-induced migration of Baf3 cells [40]. The presence of angiogenic CXCL8 in solution with the angiostatic chemokine CXCL4 increases the anti-proliferative activity of CXCL4 against endothelial cells [40]. In addition, the co-presence of CXCL4 and CXCL8, in turn, attenuates the CXCL8-mediated rise in intracellular calcium in the amyloid progenitor cell line and enhances CXCL8-induced migration of bone-marrow-derived pro-B-cells (Baf/3) [39,40]. Moreover, heterodimerization between members of the CXC and CC sub-families has also been reported with CXCL4 and CCL5 [41,42,86], as well as with CCL21 (secondary lymphoid tissue chemokine) and CXCL13 (B cell attracting chemokine-1) [25]. The functional result is that hetero-dimerization dramatically modulates biological activities of these chemokines in vitro and in vivo. In this regard, chemokine heterodimerization can modulate the overall signaling response of GPCRs, thereby providing a general mechanism for regulating chemokine function. The recent synthesis and in vitro*/*in vivo testing of a covalently-linked CXCL4/CCL5 heterodimer has validated the functional relevance of chemokine heterodimers in GPCR-mediated signal transduction [42,89].

GAGs are essential to chemokine function in vivo [54], and their structures and localization are altered after injury and during inflammation [90,91]. While the exact role of GAGs is quite diverse, it appears that chondroitin sulfate can induce a specific CCL5/CXCL4 hetero-oligomer structure that promotes atherosclerosis [41,86]. The exact molecular structure of this “active” CCL5/CXCL4 heterooligomer is unknown. The possibility of functionally relevant, structurally distinct oligomer conformations as a result of GAG/chemokine interactions has previously been postulated [47,92]. For some chemokines, homo-oligomerization is coupled to GAG binding, e.g., CCL5 and CCL3 [93]. Mikhailov et al. [23] demonstrated early on that GAG (heparin dodecasaccharide) binding to CXCL4 induces higher-order oligomer formation, dependent upon the chemokine:GAG molar ratio, which can lead to the development of thrombocytopenia. Although the role of higher-order chemokine oligomers has been widely recognized to play a role in cell signal transduction, their mechanism of action is poorly understood.

Even though there is evidence that chemokine heterodimers and/or hetero-oligomers impact biological activity, this does not exclude the occurrence of individual chemokines working in concert on their respective GPCRs to elicit synergistic effects. In fact, Gouwy et al. [94,95,96] have shown that blocking one of the two chemokine receptors negates synergistic interactions, suggesting that synergy requires each chemokine to bind to its respective cell receptor leading to intra-cellular signaling. Gouwy et al. [94] reported that CXCR4 and CCR5 ligands cooperate in monocyte and lymphocyte migration and in the inhibition of dual-tropic (R5/X4) HIV-1 infection. Gouwy et al. [96] demonstrated that chemokines and other GPCR ligands synergize in receptor-mediated migration of monocyte-derived immature and mature dendritic cells. These authors also observed synergy between chemokines and chemotactic non-chemokine GPCR agonists, including fMLP, C5a and SAA. In view of these results, it is likely that there are several ways to obtain synergy between these chemo-attractants [97].

## 4. Chemokine Antagonists

Given the important roles that chemokines and their cell surface GPCR receptors play in biology and pathological disorders, a number of chemokines and their GPCR partners have become targets for therapeutic drug development [98,99,100]. Moreover, because chemokines are major players in inflammation, pathological disorders that involve chemokines in tissue inflammation, primarily via leukocyte recruitment and activation, have been central to these efforts [101]. Various strategies have been used to intervene with chemokine systems for therapeutic purposes [102]. Knowledge of chemokine oligomer structures [103] and how chemokines interact with their receptors (see above) has been crucial to the development of chemokine antagonists. Here, we provide a limited presentation of some of these, and discuss how targeting chemokine hetero-oligomers may be another fruitful approach going forward.

One of the major strategies in this field has been the direct inhibition of GPCRs. Small molecule inhibitors targeting chemokine receptors CCR3 and CCR4 have been reported to attenuate lung inflammation in animal models [104,105,106]. A modest effect targeting CXCL8 with a mAb has been reported in a Phase II clinical trial against chronic obstructive pulmonary disease (COPD) [107]. However, this approach targeted the free CXCL8 ligand and not the ligand-receptor bound state, which is likely to be the actual “bioactive” state, thus possibly explaining the limited success using this mAb. In the rheumatoid arthritis arena, two inhibitors have shown promise: one is a CCR2/CCL2 inhibitor INCB3344 [108] and the other is a monomeric variant P8A-CCL2 antagonist [109]. However, both agents had mixed results. In fact, anti-CCR2 depleting antibody MC-21 actually exacerbated the disease [110], and three phase II clinical trials using an anti-MCP-1 antibody, an anti-CCR2 antibody, and a CCR2 inhibitor [111] all failed.

Overall, the development of highly effective chemokine antagonists has been slow. Part of the problem is that most chemokine/receptor interactions are not very selective [60,112], and thus there has been limited success in the clinic. Nevertheless, recent success with the anti-HIV drug Maraviroc has underscored the therapeutic value of interfering with chemokines in receptor binding and cell signaling [113]. There are some other exceptions that have shown potential. For example, the orally active, small molecule inhibitor CCX282-B, which targets CCR9, has shown efficacy in a colitis model in animals [114], as well as promising results in clinical trials against colitis. CCR5 and CXCR3 are central to alloimmune responses and thus are potential targets for post-transplant immunosuppression, and another small molecule antagonist of CCR5 (TAK-779) has been shown to prolong allograft survival in transplant models by attenuating recruitment of CD4^+^, CD8^+^ and CD11^+^ cells and to attenuate development of chronic vasculopathy, fibrosis, and cellular infiltration [115].

Using a quite different approach, others have demonstrated that DNA viruses can control the immune response to infection by expressing chemokine ligand [116,117] and receptor [118] homologs, as well as small chemokine-binding compounds [119]. Phage display has been used to identify the pharmacophore of CC chemokine-binding proteins Evasin-1 and -4 with the goal of developing novel drugs to target this system [120]. Another interesting approach is to “lock” a chemokine ligand into its homodimer structure, as exemplified by CXCL12 [121]. Due to enhanced serum stability, this dimeric CXCL12 variant was shown to inhibit pulmonary metastasis of CXCR4-expressing melanoma cells. CXCL12 is a popular target for drug development, as exemplified with a structure-based drug design approach leading to the development of an interesting CXCL12 antagonist [122,123]. Relatedly, Ziarek et al. [124] employed a fragment-based strategy to optimize small molecule CXCL12 inhibitors that antagonize the CXCL12/CXCR4 interaction. More recently, analysis of the CCR2 structure has led to the identification of orthosteric and allosteric antagonists of this receptor [125].

Another approach has exploited GAG–chemokine binding [126]. Evidence indicates that functional and tissue-specific selectivity are introduced into the chemokine system via the formation of chemokine oligomers that are modulated by GAG binding [54,57]. Nellen et al. [127] reported that interference with CCL5 oligomerization and GAG binding improves liver injury. Heparin oligosaccharides have been used to inhibit CXCL12-mediated cardio-protection by binding to the chemokine dimerization interface to promote oligomerization and compete with binding to the N-terminus of its receptor CXCR4 [128]. Another polysaccharide-related approach is use of a 30 kDa secreted protein, TSG-6, a member of the hyaluronan-binding protein family (hyaladherins) that contains a hyaluronan-binding LINK domain. TSG-6 inhibits neutrophil migration via direct interaction with CXCL8 [129].

Even though early reports of chemokine heterodimers [32,38,39,40] were somewhat controversial in terms of their biological relevance, this concept has been validated experimentally and does present a novel paradigm for designing chemokine antagonists [40,41,42]. Koenen et al. [41] reported on the use of CCL5-derived peptides, e.g., CKEY, that function as chemokine heterodimer agonists. The term “chemokine interactome” was recently introduced to promote the chemokine heterodimer concept and present further empirical evidence as to which CXC and CC chemokines interact physically with each other [42]. This large sampling of chemokines demonstrated that not all of them have the potential to form hetero-oligomers, which imparts selectivity to interactions between mixed chemokines. Moreover, the GAG chondroitin sulfate appears to induce a specific CCL5/CXCL4 heterodimer that enhances atherosclerotic development [41,86]. Evidence suggests that this “active” form of CCL5:CXCL4 is a promising target for therapeutic intervention selective to sites of atherosclerotic lesions [41]. Based on these studies, other chemokine-derived peptides were designed and shown to be effective chemokine antagonists [42]. However, it was the design and synthesis of a covalently-linked CXCL4/CCL5 heterodimer [89] that has provided the most compelling evidence in vitro and in vivo it validated the biological relevance of chemokine heterodimers [42]. These studies may contribute to the development and promise of novel chemokine antagonists for use in the clinic.

## Note

Given the breadth of the chemokine field and the numerous labs involved in it, we apologize for the inadvertent omission of many excellent works.
